# Macrophage migration inhibitory factor of Syrian golden hamster shares structural and functional similarity with human counterpart and promotes pancreatic cancer

**DOI:** 10.1038/s41598-019-51947-7

**Published:** 2019-10-29

**Authors:** Voddu Suresh, Rajivgandhi Sundaram, Pujarini Dash, Surendra Chandra Sabat, Debasish Mohapatra, Sneha Mohanty, Dileep Vasudevan, Shantibhusan Senapati

**Affiliations:** 1 Tumor Microenvironment and Animal Models Lab, Institute of Life Sciences, Bhubaneswar, Odisha India; 2 Macromolecular Crystallography Lab, Institute of Life Sciences, Bhubaneswar, Odisha India; 3 Regional Centre for Biotechnology, Faridabad, Haryana India; 4 Manipal Academy of Higher Education, Manipal, Karnataka India; 5 Molecular Biology of Abiotic Stress Lab, Institute of Life Sciences, Bhubaneswar, Odisha India; 6 Department of Microbiology, Odisha University of Agriculture and Technology, Bhubaneswar, Odisha India

**Keywords:** Pancreatic cancer, Nanocrystallography

## Abstract

Macrophage migration inhibitory factor (MIF) is a pleiotropic cytokine that increasingly is being studied in cancers and inflammatory diseases. Though murine models have been instrumental in understanding the functional role of MIF in different pathological conditions, the information obtained from these models is biased towards a specific species. In experimental science, results obtained from multiple clinically relevant animal models always provide convincing data that might recapitulate in humans. Syrian golden hamster (*Mesocricetus auratus*), is a clinically relevant animal model for multiple human diseases. Hence, the major objectives of this study were to characterize the structure and function of *Mesocricetus auratus* MIF (MaMIF) and finally evaluate its effect on pancreatic tumor growth *in vivo*. Initially, the recombinant MaMIF was cloned, expressed and purified in a bacterial expression system. The MaMIF primary sequence, biochemical properties, and crystal structure analysis showed greater similarity with human MIF. The crystal structure of MaMIF illustrates that it forms a homotrimer as known in human and mouse. However, MaMIF exhibits some minor structural variations when compared to human and mouse MIF. The *in vitro* functional studies show that MaMIF has tautomerase activity and enhances activation and migration of hamster peripheral blood mononuclear cells (PBMCs). Interestingly, injection of MaMIF into HapT1 pancreatic tumor-bearing hamsters significantly enhanced the tumor growth and tumor-associated angiogenesis. Together, the current study shows a structural and functional similarity between the hamster and human MIF. Moreover, it has demonstrated that a high level of circulating MIF originating from non-tumor cells might also promote pancreatic tumor growth *in vivo*.

## Introduction

Clinically relevant animal models help in understanding the pathogenesis of different human and animal diseases and play crucial roles in developing new therapeutics against them^1^. In spite of the dominance of the mouse as an experimental model animal, the hamster has carved its niche as a potential model animal for studying many diseases and for the evaluation of therapeutic agents. Hamsters are frequently used in various disease pathogenesis studies due to the ease of handling them and the similarity to humans in disease development. Hamsters are important animal models for studying various infectious diseases of humans^2–9^. The hamster models of pancreatic and oral cancer have gained importance in their respective fields^10–15^. Moreover, these animals have also been instrumental in studying metabolic and/or inflammatory diseases like diabetes and pancreatitis^16,17^. Despite the fact that the hamster is an important clinically relevant animal model for different diseases, it is not used to its full potential. In this aspect, the non-availability of complete genetic information of these animals and lack of biological reagents like recombinant proteins and antibodies related to them are the major constraints.

Macrophage migration inhibitory factor (MIF) is a pro-inflammatory cytokine with pleiotropic functions in various pathophysiological processes^18–21^. Though initially identified as a T cell-derived cytokine that inhibits macrophage migration, its pleiotropic effects on immune cells, cancer cells, as well as non-cancerous cells made MIF more enigmatic to the researchers. Involvement of MIF in several human diseases like pulmonary hypertension, endothelial cell growth, atherosclerosis, wound healing, viral infection, many cancers including lung, colon, prostate, breast and pancreatic cancer has gained significant interest in this molecule^22^. In one of our earlier published study, we have characterized HapT1 cell line-based Syrian golden hamster tumor as a model of pancreatic cancer-associated desmoplasia, an event that plays a key role in human pancreatic cancer progression. In that study, for the first time, proteomics analysis of whole-cell lysate from hamster pancreatic stellate cells (PSCs) showed expression of macrophage migration inhibitory factor (MIF) by the cells^11^. At that particular time, due to the unavailability of information and reagents for *Mesocricetus auratus* MIF (MaMIF), we were unable to investigate the function of this molecule in the hamster model of pancreatic cancer. Hence, in the current study, our major objective was to characterize the MaMIF protein and evaluate the effect of exogenous MIF on the growth of pancreatic tumor in a syngeneic model of hamster pancreatic cancer.

In the current study, we have successfully purified recombinant MaMIF protein from a bacterial protein expression system. Our analysis showed that like human MIF, MaMIF also forms a trimer in solution. A commercially available MIF antibody raised against human MIF cross-reacts with MaMIF. We resolved the trimeric MaMIF crystal structure at 1.8 Å resolution, and the structural analysis showed multiple features in MaMIF to be similar to mouse and human MIF. Further, biochemical and cell culture-based studies using endotoxin-free MaMIF showed it’s enzymatic (tautomerase) and immunostimulatory activities, which suggest that the purified protein is biologically active. Importantly, all the biological properties of MaMIF investigated in this study were similar to human MIF. At the end, we have investigated the effect of MaMIF on the growth of HapT1 pancreatic tumor in its syngeneic host. The data clearly shows the pro-tumorigenic effect of MaMIF on the HapT1 pancreatic tumor. Taken together, the data presented in this study have unraveled multiple information regarding MaMIF, and indicate the importance of hamster as a model to investigate questions related to the role of MIF in pancreatic cancer progression.

## Materials and Methods

### Recombinant MaMIF expression, purification and Immunoblotting

Syrian golden hamster (*Mesocricetus auratus*) *Mif* open reading frame sequence (spanning residues 1–115; Supplementary Fig. 1) was PCR amplified and cloned in between NdeI and XhoI sites of a pET22b+ vector with an uncleavable C-terminal hexahistidine tag. The protein was expressed in *E. coli* BL21 (DE3) cells at an OD_600nm_ of 0.6, by induction with 0.5 mM IPTG for 4 hours at 37 °C. Cells from 1 liter culture were pelleted down by centrifugation for 10 min at 6000 × g and then suspended in 50 ml of buffer A containing 20 mM Tris-HCl (pH 7.5), 20 mM imidazole, 300 mM NaCl, 1 mM βME, 1 mM PMSF and one tablet of EDTA-free protease inhibitor cocktail (Sigma). The cells were lysed by sonication and the lysate was clarified by centrifugation at 40,000 × g for 45 min. Recombinant MaMIF was first captured on a Ni-NTA affinity column (HisTrap FF 5 ml, GE Healthcare). Then the column was washed with 15 column volumes of buffer A and eluted with a linear gradient of buffer B (buffer A supplemented with 500 mM imidazole), followed by size-exclusion chromatography using a HiLoad 16/600 Superdex 75 pg column (GE Healthcare) with buffer C containing 20 mM Tris-HCl (pH 7.5), 150 mM NaCl and 1 mM DTT. The peak fractions containing MIF were pooled and concentrated to 30 mg/ml and stored at −80 °C. The purified protein was analyzed on 18% SDS-PAGE and stained with Coomassie Brilliant Blue to confirm the purity and to get an estimate of the monomeric molecular mass. To estimate the approximate molecular mass of purified MaMIF in native conformation, *i.e*. to find whether the protein exists in an oligomeric form, analytical size-exclusion chromatography was performed using buffer C with help of Superdex 75 10/300 GL column which was pre-calibrated with Low molecular weight standards (GE Healthcare). For biochemical experiments, endotoxin was removed from purified MaMIF using Vivaspin endotest tubes (Sartorius Biotech, Germany). Next, the *E. coli*-derived recombinant MaMIF used in our biochemical experiments was used to perform Western blot to elucidate the cross-reactivity of MIF polyclonal antibody (NBP1-81832; Novus Biologicals) towards MaMIF, since this antibody was raised against a recombinant protein with 100% identity with human MIF and 89% identity with MaMIF. In parallel, a non-MIF primary antibody with similar isotype (anti-Bak antibody, Rabbit mAb #12105, CST) was used to detect the background level of MaMIF detection.

### CD spectroscopy

CD spectra of 10 μM MaMIF in a buffer containing 20 mM Tris-HCl (pH 7.5) and 50 mM NaCl were recorded using a Chirascan CD spectroscope (Applied Photophysics). The spectra were collected in triplicate from 260 nm to 190 nm at 25 °C at a bandwidth of 1.0 nm using a quartz cuvette with 10 mm path length. All the spectra were averaged and the buffer spectrum was subtracted from those of sample. The estimation of secondary structural elements was done using the BeStSel server^23^. The CD intensities (in millidegrees) are plotted against wavelength.

### Multiple sequence alignment and phylogenetic tree analysis

Protein sequences of Syrian golden hamster MIF (UniProtKB: A0A140EDM8), mouse MIF (UniProtKB: P34884) and human MIF (UniProtKB: P14174) were aligned using PRALINE multiple sequence alignment program and residue substitution matrix BLOSUM62 at http://www.ibi.vu.nl/programs/pralinewww/. PRALINE sequence conservation score 0 was given for the least conserved position and 10 for the most conserved position of alignment. The phylogenetic tree was constructed with MEGA 7 software^24^ using the Neighbor-Joining method^25^. The optimal tree with the sum of branch length is 0.15113287 and was drawn to scale with branch lengths (below the branches) in the same units as those of the evolutionary distances used to infer the phylogenetic tree. The evolutionary distances were computed using the Poisson method in the units of several residue substitutions per position.

### Crystallization and data collection

Recombinant MaMIF (15 mg/ml) was screened for crystallization at 18 °C using commercially available screens from Hampton Research with the help of a Formulatrix NT8 crystallization robot in 96-well sitting drop crystallization plates. After 3 days, small and two-dimensional crystals appeared in a few conditions. The sequential micro-seeding approach was adopted for further optimization. Towards this end, crystals grown in Hampton Research PEG/Ion HT F8 condition containing 50% v/v PEG 400, 200 mM lithium sulfate and 100 mM sodium acetate (pH 7.5) were crushed by using the seeding kit (Hampton Research) and used for micro-seeding into the same condition, but in bigger drops with 1 μl protein and 1 μl condition, using hanging drop vapor diffusion method. After a week, bigger crystals appeared but were still two-dimensional in morphology. These crystals were picked, crushed and used as seed material for fresh crystallization screens following the random micro-seed matrix-screening method^26^ in 96-well sitting drop plates, with the help of an NT8 crystallization robot. Crystals appeared in several new conditions; however, most of them were still two-dimensional and plate-like in morphology. A condition having 200 mM succinic acid (pH 7.0) and 20% w/v PEG 3350 yielded a bigger and thicker crystal after three weeks. The crystal was flash cooled in liquid nitrogen, after transferring to a solution with the composition of the crystallization condition, supplemented with 20% ethylene glycol. The diffraction images were recorded on an Eiger X 4M detector (DECTRIS) on beamline ID30A-3 (MASSIF-3) of European Synchrotron Radiation Facility (Grenoble, France). 2000 images were collected with an oscillation of 0.1°, at a wavelength of 0.9677 Å.

### Data processing, structure determination and refinement

The diffraction images were processed using XDS^27^. The crystal structure of MaMIF was solved by the molecular replacement method with the help of the program Molrep^28^ from the CCP4 suite^29^, using the mouse MIF crystal structure (PDB id: 1mff) as the search model. Crystallographic refinement and model building were performed iteratively using Refmac5^30^ and Coot^31^. The quality of the final model was assessed using the Ramachandran Plot from the programs PROCHECK^32^ and Molprobity^33^. All the figures were prepared using PyMOL (Schrödinger, LLC). The data collection and refinement statistics are provided in Table 1. The structure factors and refined coordinates have been deposited in the PDB, with the accession code 6ice.

**Table 1:** Crystal data collection and refinement statistics.

Parameter	MaMIF
**Data Collection**	
Beamline	ESRF-MASSIF-3
Detector type	Dectris Eiger X 4M
Wavelength (Å)	0.9677
Data collection temperature (K)	100
Space group	P2_1_2_1_2_1_
a, b, c (Å)	53.88, 55.85, 112.16
α, β, γ (°)	90, 90, 90
Resolution (Å)	56.08–1.80 (1.84–1.80)
Rmeas (%)	0.118 (0.599)
I/σ(I)	11.8 (3.5)
CC (1/2) (%)	0.997 (0.868)
Total number of reflections	239343 (14289)
Mosaicity (°)	0.65
Completeness (%)	98.5 (99.7)
R_merge_	0.103 (0.525)
Multiplicity	7.6 (7.8)
Wilson B-factor (Å^2^)	6.78
Matthews coefficient (Å^3^/Da)	2.14
Solvent content (%)	42.46
No. of molecules in ASU	3
**Refinement**	
No. of unique reflections	31590 (1840)
R_work_/R_free_ (%)	17.6/21.3
No. of protein atoms	2587
No. of solvent atoms	196
Mean B-factor (Å^2^)	15.75
**R.m.s. deviations**	
Bond lengths (Å)	0.020
Bond angles (°)	1.807
**Ramachandran plot (%)**	
Preferred region	99.10
Allowed region	0.90
Outliers	0.0

Numbers in parentheses correspond to the last resolution shell.

### Tautomerase assay

MIF from other species has been reported to possess one unusual activity of catalyzing the tautomerization of D-dopachrome and L-dopachrome methyl ester into their corresponding indole derivatives^34^. Tautomerase assay was carried out as described previously^35^. To analyze the tautomerase activity of MaMIF, 4 mM of L-3,4-dihydroxyphenylalanine methyl ester was diluted in 5 ml autoclaved, double-distilled water and an appropriate amount of sodium periodate was added to it to make a final concentration of 8 mM. The mixture was incubated on ice for 20 min in the dark. 300 μl from this solution was mixed with 700 μl of the assay buffer consisting of 50 mM potassium phosphate and 1 mM EDTA (pH 6.0). Increasing concentrations of MIF were added to it and the decrease in absorbance at 475 nm was recorded after different time points of incubation. To confirm the specificity of MIF tautomerase activity, a similar experiment was conducted with BSA as a control.

### Effect of MaMIF on the expression of inflammation-associated genes in hamster peripheral blood mononuclear cells (PBMCs)

To check the effect of MIF on the expression status of certain inflammation-associated genes in PBMCs, cells were isolated from hamster blood using the Histopaque gradient as suggested by the manufacturer (Sigma, USA). The isolated PBMCs were cultured in 10% RPMI media and treated with BSA (100 ng/ml), MaMIF (100 ng/ml)^36–38^, Polymyxin B (30 μg/ml), MaMIF + Polymyxin B, ISO-1 ((S, R)-3-(4-hydroxyphenyl)-4, 5-dihydro-5-isoxazole acetic acid methyl ester) (100 μg/ml) or MaMIF + ISO-1 for 4 hours. Then cells were harvested in RLT buffer and processed for RNA isolation as described by the manufacturer (Qiagen, USA). RNA quantification was carried out using Nanodrop followed by cDNA synthesis^11^. The expression levels of *Tnf-α Il-6*, *Il-1β* and *Vegf*, and were analyzed in all the cDNA samples through qPCR, by using gene-specific primers. Polymyxin B was used to neutralize any residual LPS present along with MaMIF protein.

### Effect of MaMIF on PBMC migration

Prior approval from the Institutional Animal Ethical Committee (Institute of Life Sciences, Bhubaneswar, India) was taken for all the protocols used in the animal studies. All the methods associated with animal studies were performed according to the Committee for the Purpose of Control and Supervision of Experiments on Animal (CPCSEA), India guidelines. Preparation of hamster peripheral blood mononuclear cells was carried out as previously described^39^. Briefly, normal hamsters having no symptomatic diseases were euthanized and blood was collected through the cardiac puncture in a heparinized tube followed by Histopaque (Sigma, 11191) gradient centrifugation. After gradient centrifugation, the cells present in the buffy coat layer were aspirated and washed twice with 10 ml PBS followed by resuspension in 2 to 3 ml of 0% FBS containing RPMI media. Cell counting was done through the trypan blue dye exclusion method. To check the effect of recombinant MaMIF on the migration of hamster PBMCs, 3.5 × 10^5^ PBMCs in 0% FBS containing media (500 µl) were seeded in 8 µm pore trans-well insert (Millicell Hanging Cell Culture Insert, PET 8 µm, 12-well, MCEP12H48) and added either BSA (100 ng/ml), MaMIF (100 ng/ml), MaMIF (100 ng/ml) + Polymyxin B (30 μg/ml) or Polymyxin B (30 μg/ml) in 1% FBS containing media, as chemoattractant (600 µl) to the lower chamber. Polymixin B, was used to neutralize any endotoxin contamination, if present in the purified MaMIF^40^. Each experimental condition was carried out in triplicate. After 2 hours of incubation at 37 °C and 5% CO_2_, the cells were fixed in 10% buffered formalin for 10 min followed by staining with 0.6% crystal violet. Cells adhered to the upper surface of the membrane were removed with a cotton swab. Migrated cells at the lower surface of the insert were visualized under an inverted bright field microscope and 5 to 8 random images were captured for each membrane. The numbers of migrated cells were then expressed as the average of total field per membrane in triplicate for each experimental condition. The gene expression analysis and PBMC migration experiments were repeated minimum of three times in triplicate. Although we noticed slight variations between the experimental values, the resulting pattern was quite similar. The data provided in the manuscript represents data obtained from one set of experiments.

### Effect of MaMIF on pancreatic tumor growth *in vivo*

Syrian golden hamsters (3–4 months old; male) were inoculated subcutaneously with 4 × 10^5^ HapT1 pancreatic cancer cells. Animals were checked for palpable tumors on a daily basis. After six days from the injection of cancer cells, the tumor-bearing animals were randomized into two groups (n = 5 per group) and treated with MaMIF dissolved in PBS (1 mg/kg) or PBS intraperitoneally for up to 13 days post-inoculation of tumor cells. Tumor dimensions were measured every day, and tumor volume was calculated by using the formula V = (W^2^ × L)/2 for caliper measurements where V is the tumor volume, W is the tumor width and L is the tumor length. All the animals were sacrificed on the 20^th^ day and tumors were harvested. After measuring the tumor weights, the samples were preserved and processed for histopathological analysis.

### Histology and immunohistochemistry

HapT1 tumors were dissected and fixed in 10% neutral buffered formalin at room temperature for 48 hours, and then tumor tissues were dehydrated and embedded in paraffin wax. 5 µm thick sections were cut and stained with hematoxylin-eosin. For immunohistochemistry, 5 μm paraffin-embedded sections were de-paraffinized in xylene and rehydrated with graded ethanol and deionized water, then boiled in acidic pH citrate buffer (Vector Laboratories) for 20 min in a steam cooker. Endogenous peroxidase was blocked with 3% hydrogen peroxide in methanol for 20 min, and then washed with PBS followed by blocking with horse serum (Vector Lab) for 30 min. Then sections were incubated with the anti-Ki67 antibody (VP-RM04, Vector Laboratories) overnight at 4 °C in a humidified chamber followed by washing with PBS. Then slides were incubated for 45 min at room temperature with a horse anti-rabbit/mouse IgG biotinylated universal antibody (Vector Laboratories) and then with ABC reagent for 30 min. The stain was developed with 3, 3′-diaminobenzidine (DAB; Vector Laboratories) substrate according to the manufacturer’s instructions. The slides were counter-stained with hematoxylin, dehydrated, cleared and mounted with mounting media (Vector Laboratories). Slides were observed under a Zeiss ApoTome.2 microscope and images captured at 10x, 20x and 40x magnifications. For quantification of blood vessels, multiple images from different areas of Haematoxylin & Eosin (H&E) stained HapT1 tumor sections were digitally captured and the number of blood vessels/capillaries per field were counted.

### Effect of MaMIF on the overall growth of HapT1 cells *in vitro*

To check the effect of MaMIF on the proliferation of HapT1 cells, 20,000 cells were seeded into the 24-well plate and allowed for attachment. Then, treatment with MaMIF in different concentrations (0, 10, 50, 100, 150, 200, 300, 400 ng/ml) was given in triplicate to each group. After 48 hours, cells were washed twice with PBS and then fixed in 10% neutral buffered formalin for 10 min and stained with 0.6% crystal violet solution for 30 min, followed by de-staining with water. Quantification was done by dissolving crystal violet in 10% acetic acid for 15 min. Absorbance was measured at 570 nm using a Varioskan Flash Multimode Reader (Thermo Scientific).

To check the effect of ISO-1 on HapT1 cells growth, 5000 cells/well were seeded in 24-well plates and allowed to adhere for 24 hours at 37 °C and 5% CO_2_. Cells were treated with MaMIF (100 ng/ml), ISO-1 (100 µg/ml) or MaMIF + ISO-1 in triplicate for each group. After 48 hours, MTT (5 mg/ml) was added to cells and incubated for 4 hours. The media was discarded and all the precipitates were dissolved in 250 µl DMSO to measure the absorbance at 570 nm using Varioskan Flash Multimode Reader (Thermo Scientific).

### Effect of MaMIF on VEGF expression in HapT1 cells

To check the effect of MaMIF on the expression status of VEGF in HapT1 cells, 1,00,000 cells were seeded into 60 mm petri plate. After cells got attached, treatment was given to different groups such as control, MaMIF (100 ng/ml), ISO-1 (100 µg/ml) and MaMIF + ISO-1. After 48 hours of the treatment, cells were processed and RNA isolated using RNA isolation kit (Qiagen, USA). RNA quantification was done using Nanodrop spectrophotometer (Thermo Scientific) followed by cDNA synthesis. The expression status of *Vegf* was analyzed with the help of qPCR, by using the following specific primers: Forward: CTGGCTGGGTCACTAACA; Reverse: TTCTGGCTTTGTTCTGACTT.

### Knockdown of MaMIF by siRNA transfection

Small interfering RNA (siRNA) oligonucleotides targeting MaMIF were purchased from Eurogentec. The sequence of Sense siRNA was as follows: 5′-UAAUAGUUGAUGUAGAUCCGG-3′ and that of the Anti-sense siRNA was 5′-CCGGAUCUACAUCAACUAUUA-3′. 20,000 HapT1 cells were seeded in 24-well plates and cultured for 24 hours with MEM Eagle medium (PAN Biotech). Cells were transfected with the scramble and MIF siRNA having a final concentration of 10 nM using Lipofectamine RNAiMAX transfection reagent (Invitrogen) according to the manufacturer’s instructions. After 48 hours of transfection, cells were harvested for qRT-PCR. In parallel, the corresponding culture plates were processed for crystal violet staining and quantification. *In vitro* viability experiments were performed with minimum of three technical, as well as three experimental replicates and the data shown is an average of the averages (n = 3).

## Results

### Expression and purification of recombinant MaMIF

**Figure 1:**
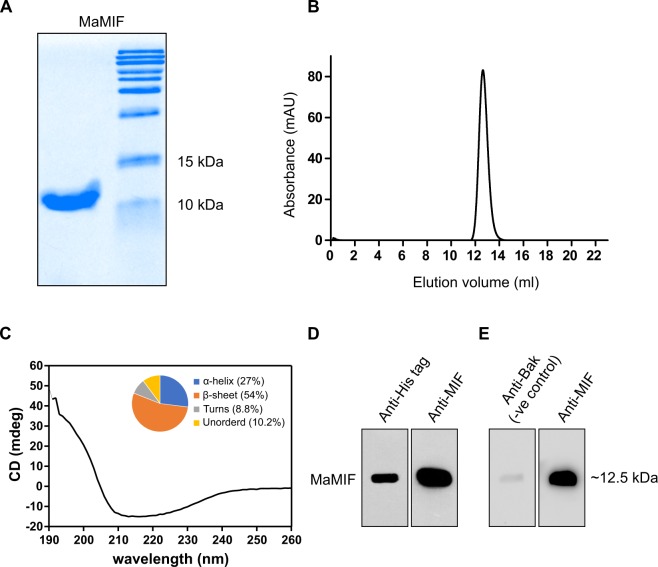
Expression and purification of MaMIF. (**A**) SDS-PAGE analysis of purified MaMIF suggesting an approximate molecular mass of ~12.5 kDa for the monomer. (**B**) Superdex 75 10/300 GL analytical size-exclusion chromatography profile of MaMIF suggesting an apparent molecular mass of ~39 kDa in solution, corresponding to the size of a trimeric form. (**C**) CD spectrum of MaMIF confirming a properly folded protein. The pie chart in the inset shows the secondary structure make-up of the protein. (**D**) Western blot analysis with polyclonal anti-His and anti-MIF antibodies confirming the expressed protein to be MaMIF. (**E**) Western blot analysis with isotype-matched primary antibody shows the background level of antibody binding to the protein.

Although MIF was the first described cytokine, its role in some important pathophysiological conditions across different species have remained unclear. MIF from human and mouse or even from different human parasites have been structurally and functionally characterized^41–43^. In this study, we cloned and purified MaMIF as described in the materials and methods section. In one of our previous studies, we had cloned MaMIF coding sequence (CDS) from mRNA isolated from hamster pancreatic stellate cells^11^. The mouse genome is known to harbor multiple MIF-related sequences; however, only the true *Mif* gene has an intron/exon structure and a 5′ untranslated region^44^. Although the mouse MIF-related pseudogenes are highly homologous to cDNA, those contain different mutations that would generate truncated or altered MIF-like proteins. Hence, before cloning the hamster *Mif* CDS into the expression vector, we checked the hamster *Mif* genome organization. *In silico* analysis of recently updated genome sequence available in NCBI database (NW_004801628.1) confirmed that the *Mif* CDS cloned in this work is from the true *Mif* gene, which has a similar genomic organization as the human and mouse genes (Supplementary Fig. 1). The expressed and purified MaMIF showed an approximate molecular mass of 12.5 kDa with more than 95% purity as observed from 18% SDS-PAGE, stained with Coomassie Brilliant Blue (Fig. 1A). Comparison of the elution volume of MaMIF with low molecular weight protein standards in the analytical size-exclusion chromatographic experiment suggested the molecular mass of MaMIF in solution to be approximately three-times higher (39 kDa) than the monomer mass (12.5 kDa). This result indicates that the purified recombinant MaMIF exists as a trimer (Fig. 1B), like human and mouse MIF. The CD spectroscopic analysis showed the recombinant protein to be properly folded. The secondary structure content of the protein was estimated to be 27% α-helix, 54% β-sheet, 8.8% turns and 10.2% unordered structure, with an RMSD and NMRSD values of 0.40 and 0.0237, respectively, as given in the form of a pie chart along with the profile (Fig. 1C). The Western blot analysis revealed the cross-reactivity of MIF polyclonal antibody (NBP1-81832; Novus Biologicals) towards MaMIF and further confirmed successful expression and purification of the protein (Fig. 1D). Immunoblot analysis using a non-MIF primary antibody with similar isotype (rabbit IgG) as a negative control showed very minimal background signal compared to the MIF-specific primary antibody (Fig. 1E).

### Sequence and phylogenetic analysis of MaMIF

**Figure 2:**
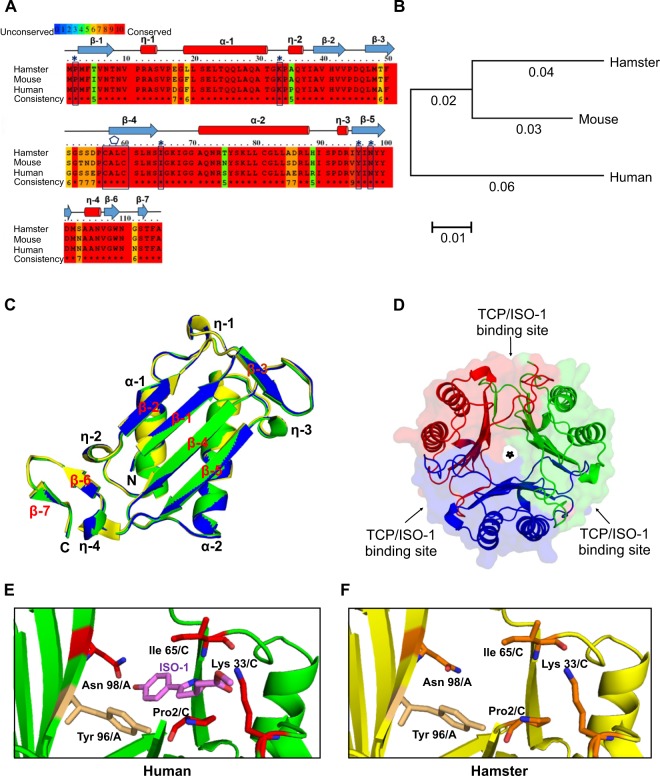
Syrian golden hamster MIF protein sequence and structural analysis. (**A**) Primary sequences of MIF from the Syrian golden hamster (UniProt accession number A0A140EDM8) mouse (P34884) and human (P14174) were aligned with PRALINE program. The conserved residues are shown in red color. The boxes with cap indicate the conserved residues of the tautomerase active site, and the polygon label above the boxed sequence shows the conserved ‘CALC’ motif responsible for the oxidoreductase activity. Secondary structural elements of MaMIF are indicated above the aligned sequences. (**B**) Phylogenetic tree of hamster, mouse and human MIF constructed using MEGA 7.0 software. The branch-lengths indicate the evolutionary distances. (**C**) Superposition of the monomeric structure of MIF from hamster (yellow; PDB id: 6ice, from this work), mouse (blue; PDB id: 1mfi) and human (green; PDB id: 3djh) in cartoon representation, revealing similar 3D-structure. The MaMIF structure aligns with human MIF and mouse MIF with RMSDs of 0.24 Å and 0.26 Å, respectively for the Cα atoms. (**D**) Crystal structure of homo-trimeric MaMIF in cartoon representation, along with semi-transparent surface representation. The monomers are colored in red, green and blue. The three tautomerase catalytic pockets (TCP; also, the sites for ISO-1 binding) located at the monomer-monomer interfaces are labeled with arrows and the central solvent-accessible channel is marked with a star sign. (**E**) A magnified view of the tautomerase catalytic pocket (TCP) of the ISO-1 bound human MIF structure (PDB id: 1ljt) in green, wherein the active site residues Pro2, Lys33, Ile65, and Asn98 that are interacting with ISO-1 are colored in red and Tyr96 which is part of the catalytic pocket, and bind to ISO-1 via π-interaction is colored in light orange; all in stick representation. ISO-1 is shown in stick representation, in magenta color. (**F**) A similar magnified view as (**E**) for MaMIF TCP from this work, wherein the structure is shown in yellow, the active site residues Pro2, Lys33, Ile65, and Asn98 are shown in orange and Tyr96 in light orange; all in stick representation.

Alignment of the primary sequences of MIF from hamster, mouse, and human was performed with PRALINE program. Figure 2A indicates that MaMIF shares 93% and 89% sequence identity with mouse MIF and human MIF, respectively. The alignment reveals that the residues responsible for tautomerase activity and substrate binding such as Pro2, Lys33, Ile65, Tyr96, and Asn98 are conserved in MaMIF as well. In addition, the ‘CALC’ motif required for oxidoreductase activity is also conserved in MaMIF (Fig. 2A). As both the tautomerase and oxidoreductase sites are present in hamster MIF, it might have similar enzymatic and biological activities as that of human and mouse MIF. The phylogenetic analysis of MIF from hamster, mouse and human was carried out using the Neighbor-Joining method with the help of MEGA 7 software further confirmed that MaMIF is evolutionarily closer to the mouse MIF than human MIF (Fig. 2B).

### Crystal Structure of MaMIF

The data collection and refinement statistics are given in Table 1. The crystal structure of MaMIF was resolved to 1.8 Å resolution. It forms a trimer, with three identical molecules in an asymmetric unit. A monomer of MaMIF is made up of six beta strands, four of which form a mixed beta sheet within one subunit, two antiparallel alpha helices and two shorter beta strands on either end of the sheet (Fig. 2C). Also, a very small beta strand (β7) is present towards the C-terminus. The monomer structure is highly conserved among MIF from human, mouse and hamster, as could be observed from the structure alignment (Fig. 2C). The RMSD for the Cα backbone atoms between hamster and human MIF (PDB id: 3djh) and between hamster and mouse MIF (PDB id: 1mfi) are 0.24 Å and 0.26 Å, respectively. The trimer adopts a barrel-like architecture formed by three inter-connected monomeric subunits (Fig. 2D), as has been reported for other MIF structures. The trimeric MaMIF structure is retained by the short beta strands β3 of one monomer and β6 of the third monomer becoming part of the beta sheet formed by the beta strands β1, β2, β4 and β5 of the second monomer in between, together forming a six-stranded beta sheet. Herein, β3 of monomer-1 comes in close proximity and form hydrogen-bond network, typical of beta sheets, with β2 of the monomer-2 and β6 of monomer-3 comes close to β5 of monomer-2. This contribution of beta strands by all three monomers to their respective adjacent monomers to form beta sheets seems to provide stability to the trimeric structure. In the trimeric ring structure of MIF, the beta sheets of the three monomers together form a solvent-accessible central water channel, whose role in the context of MIF function is still not known^41^. A MIF trimer has been reported to have three catalytic pockets for tautomerase activity, located in the monomer-monomer interface between the three monomers (Fig. 2D). The residues Pro2, Lys33 and Ile65 from one monomer and the residues Tyr96’ and Asn98’ from its adjacent monomer^45^, together form the catalytic pocket responsible for the tautomerase activity in human and mouse MIF and these residues are conserved in MaMIF as well. This would explain why MaMIF also shows tautomerase activity (described in the next section). Tautomerase activity of human MIF is known to be inhibited by the MIF-inhibitor ISO-1^46^. ISO-1 binds to the residues Pro2, Lys33, Ile65, Tyr96, and Asn98^47^, all of which form part of the tautomerase catalytic site located at the interface of two monomers (Fig. 2E, Supplementary Fig. 2). The aromatic ring of human MIF Tyr96 and the hydroxyphenyl ring of ISO-1 make a π-interaction, wherein, the angle between the ring planes is 78° and the distance (edge-to-face) between the ring centroids is 4 Å. As the residues of the catalytic pocket and ISO-1 binding are completely conserved in MaMIF (Fig. 2F), ISO-1 and other similar compounds binding to the catalytic pocket would block MaMIF catalytic function as well. Several reported MIF structures have sulfate ions bound to the positively charged residues lining the central solvent channel (involved in transporting the negative ions/ligands) or to the peripheral helix of MIF^48–50^. However, the MaMIF structure from this work did not have any sulfate ions, presumably because the final crystallization condition did not have any sulfate.

### Tautomerase activity of MaMIF

Unlike all other cytokines, MIF has a tautomerase activity, and it performs this enzymatic function through an N-terminal catalytic proline base^51^. In this study, the tautomerase activity of the purified MaMIF was analyzed using L-dopachrome methyl ester substrate. The activity was determined after background correction for the non-enzymatic activity (dopachrome dependent non-specific activity; Supplementary Fig. 3A,B). As the linear range of the kinetic assay for the L-dopachrome methyl ester is too short, the slope value from the initial linear part (ranging from 0 to 15 sec.) of the declining curve (Δ abs. s^−1^) was considered to determine the activity (Supplementary Fig. 3C). Supplementary Fig. 3D shows that BSA by itself, which was used as a negative control has no tautomerase activity. However, the addition of MaMIF to the BSA showed enzymatic activity.

**Figure 3:**
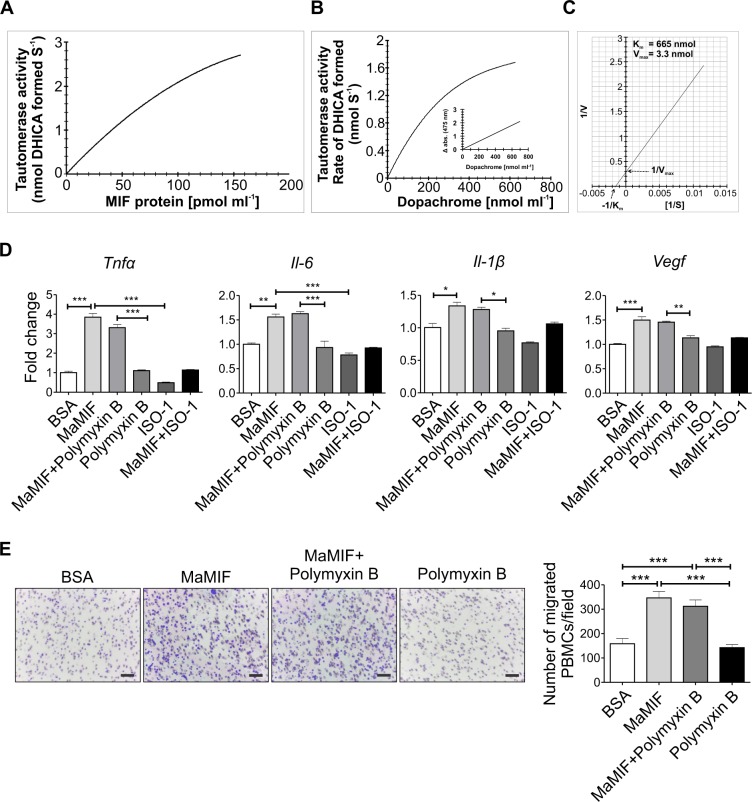
*In vitro* functional analysis of MaMIF. (**A**) Data shows the MaMIF-tautomerase activity under the increasing concentration of this protein. (**B**) Graphical representation showing the influence of increasing concentrations of dopachrome on MIF-tautomerization activity. (**C**) Determination of the K_m_ and V_max_ for the substrate-dependent MIF-tautomerase activity. (**D**) Figure shows the expression of *Tnf-α*, *Il-6*, *Il-1β,* and *Vegf* in PBMCs following stimulation with BSA (100 ng/ml), MaMIF (100 ng/ml), Polymyxin B (30 μg/ml), MaMIF + Polymyxin B, ISO-1 (100 μg/ml) and MaMIF + ISO-1 for 4 hours (n = 3). Data represents the mean ± SEM, *p < 0.05; **p < 0.001; ***p < 0.0001 using one-way ANOVA with Bonferroni’s Multiple Comparison Test. (**E**) Results of the trans-well migration assay show the effect of MaMIF on the migration property of PBMCs. Digital images of the stained trans-well porous membrane show PBMCs (blue colored) that have migrated through the pores after treatment with BSA (100 ng/ml), MaMIF (100 ng/ml), Polymyxin B (30 μg/ml), MaMIF + Polymyxin B, ISO-1 (100 μg/ml) and MaMIF + ISO-1 for 2 hours. Quantification of the number of migrated cells in different conditions is shown through the bar graph (n = 3). Each bar represents the mean ± SEM, ***p < 0.0001 using a Student t-test. The scale bar is 100 µm.

The rate of tautomerase activity of the protein (*i.e*., the amount of the product formed per second) showed a typical enzymatic response, wherein activity increased with increasing concentration of protein at lower concentrations and revealed a saturation tendency at higher concentrations (Fig. 3A). The initial velocities of the reaction were measured as a function of substrate concentration from 0 to 800 μM (nmol ml^−1^). The data presented in Fig. 3B was used to determine the K_m_ by non-linear least-squares fit method following a simple Michaelis-Menten equation (Fig. 3C). The calculated K_m_ value is 665 μM (nmol ml^−1^).

### Effect of MaMIF on activation and migration of PBMCs

MIF is known as a potent pro-inflammatory cytokine and regulates activation and migration of immune cells^52^. Exogenous MIF treatment induces expression of various pro-inflammatory cytokines (e.g. TNF-α, IL-6, IL-1β, and IL-8) and angiogenic factors (e.g. VEGF) in different immune and/or endothelial cells^53–55^. In this study, we investigated the effect of endotoxin-free MaMIF on the activation of hamster PBMCs in the presence and absence of ISO-1. Even though we removed endotoxin using Vivaspin endotest tubes, we further eliminated the chance of any residual endotoxin presence by neutralizing with polymyxin B (30 µg/ml)^56^. As a readout of PBMC activation, the level of *Tnf-α*, *Il-6, Il-1β*, and *Vegf* expression was measured through quantitative PCR analysis. Exposure to exogenous MaMIF induced significantly higher expression of *Tnf-α* (p < 0.0001)*, Il-6* (p < 0.001), and *Vegf* (p < 0.0001) in PBMCs and the addition of polymyxin B did not alter these effects of MaMIF (Fig. 3D). The tautomerase activity of MIF is known to induce cytokine expression in PBMCs and ISO-1 partially inhibits the MIF induced cytokine expression^57^. Except for *Vegf*, the MaMIF-induced expression of other factors (*Il-1β*, *Il-6*, and *Tnf-α*) got considerably suppressed upon ISO-1 treatment (Fig. 3D). Interestingly, ISO-1 itself suppressed the endogenous level of *Tnf-α, Il-1β,* and *Il-6* in PBMCs, which indicates the role of endogenous MIF in regulating the expression of these factors in PBMCs (Fig. 3D). Effect of different cytokines on immune cell activation and migration/recruitment together contribute to the overall inflammation at the site of injury and/or infection. Human MIF is known to act as a chemoattractant for PBMCs^52^. Hence, we wanted to check whether MaMIF has a similar effect on the migration properties of PBMCs. The results of our trans-well migration assay showed a significantly higher number of migrated PBMCs (p < 0.0001) in conditions where MaMIF was used as a chemoattractant as compared to BSA (Fig. 3E).

### Effect of MaMIF on the growth of HapT1 pancreatic tumor in its syngeneic host

**Figure 4:**
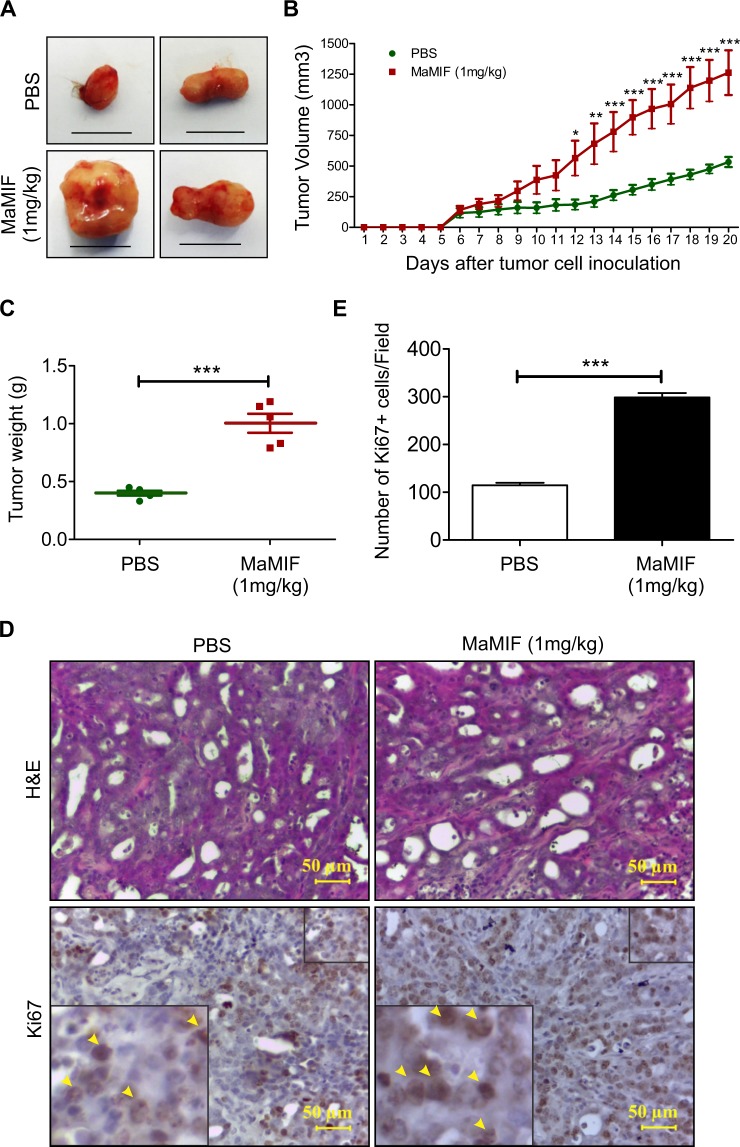
Effect of MaMIF on HapT1 tumor growth in its syngeneic host. (**A**) Representative digital images of HapT1 tumors obtained from PBS or MaMIF injected animals show bigger tumor mass in MaMIF injected groups. The scale bar is equivalent to 1 cm. (**B**) Graphical representation of tumor volume calculated every day after initiation of treatment revealed a significant increase in the tumor growth in MaMIF injected animals (n = 5). Data represents the mean ± SEM, ***p < 0.0001 using two-way ANOVA. (**C**) The dot-plot graph showing a significant increase in HapT1 tumor weight in MaMIF treated animals than control (n = 5). Data represents the mean ± SEM, ***p < 0.0001 using a Student t-test. (**D**) Hematoxylin and Eosin (H&E) stained HapT1 tumor tissue sections show moderately differentiated tumors in all the conditions (PBS or MaMIF injected). The lower panel representative images show tissue sections stained with Ki67 antibody. Images were captured at 40× magnification. (**E**) Graph showing the number of Ki67 positive cells/field (n = 5). Data represents the mean ± SEM, ***p < 0.0001 using a Student t-test.

In pancreatic cancer patients, the tumor tissues and circulating blood have a higher level of MIF than in healthy subjects^58,59^. Pancreatic cancer cells overexpress MIF and are believed to be the major contributor to the overall MIF level in the pancreatic tumor microenvironment. At the same time, multiple normal cells like immune cells, mesenchymal stem cells, endothelial cells, etc. also express MIF^60^. To understand the pathophysiological role of MIF in pancreatic cancer progression, most of the studies have adapted genetic and chemical targeting of MIF expressed by the cancer cells. However, in these approaches, the effect of MIF produced by normal cells on cancer cells (paracrine effects) is not being addressed. Hence, in the current study, we evaluated the impact of MaMIF on the growth of HapT1 tumors in its syngeneic host. The data in Fig. 4A,B shows that systemic administration of MaMIF significantly enhanced HapT1 tumor growth. Though tumor uptake was 100% in both PBS and MaMIF injected groups, but after 20 days of cancer cells injection, the tumor weight of MaMIF injected tumors was significantly higher (p < 0.0005) than PBS injected tumors (Fig. 4C). Moreover, the presence of a substantially higher number of Ki67-positive cells (p < 0.0001) in the tumors of MaMIF-injected animals than PBS injected animals (Fig. 4D,E) corroborates the observed pro-tumorigenic effect of exogenous MIF on pancreatic tumors *in vivo*.

### Mechanistic aspects of MaMIF-mediated HapT1 tumor growth in its syngeneic host

**Figure 5:**
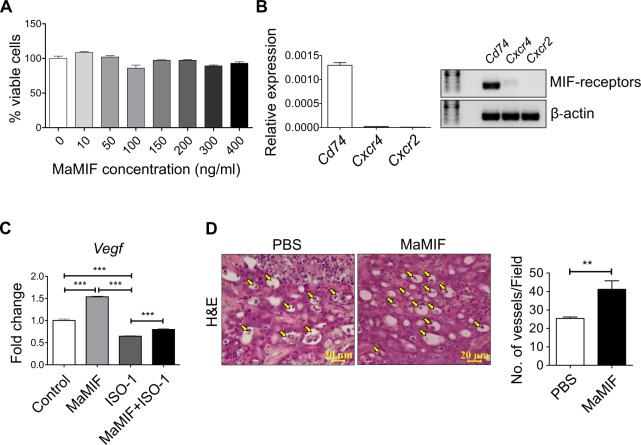
Effect of MaMIF on HapT1 cells growth and angiogenesis. (**A**) Graph showing the percentage of viable cells upon treatment with different concentrations of MaMIF after 48 hours. (**B**) Graph showing the relative expression of Cd74, Cxcr4 and Cxcr2 in HapT1 cells and agarose gel image showing the amplified products in qPCR. Relative expression is calculated using formula 2^−dCT^, dCT = CTgene − CTβ-actin. (**C**) Graph showing the expression of *Vegf* in HapT1 cells following stimulation with MaMIF (100 ng/ml), ISO-1 (100 µg/ml) and MaMIF + ISO-1 for 48 hours (n = 3). Each bar represents the mean ± SEM. ***p < 0.0001 using one-way ANOVA with Bonferroni’s Multiple Comparison Test. (**D**) Representative H&E images (captured in 40x magnification) showing the number of blood vessels (arrows indicated) in PBS and MaMIF treated tumor tissues. Quantification was done by counting the number of vessels/field in 20x magnification images. Each bar represents the mean ± the SEM. **p < 0.001 using a Student t-test.

Overexpression of MIF by cancer cells is known to promote tumor growth through multiple mechanisms^61^. To check the direct effect of MaMIF on HapT1 cancer cells growth, we treated HapT1 cancer cells with MaMIF *in vitro* and after 2 days, the number of viable cells was quantified through crystal violet staining of the cultured cells. The data showed in Fig. 5A shows that exogenous MaMIF does not have a significant effect on the overall growth of HapT1 cells *in vitro*. This result prompted us to check the status of MIF receptor expression in HapT1 cells, and our quantitative PCR analysis data clearly shows a high level of Cd74 and low level of Cxcr4, and Cxcr2 expression in HapT1 cells (Fig. 5B). At the molecular level, to check the response of HapT1 cells to exogenously treated MaMIF, we checked the status of *Vegf* expression in MaMIF-treated and -untreated HapT1 cells. The gene expression analysis showed a significant up-regulation in *Vegf* expression upon MaMIF treatment (Fig. 5C). MIF is known to induce tumor angiogenesis in different cancers^62^. Moreover, the up-regulation of *Vegf* in HapT1 cells upon MaMIF stimulation suggests that MaMIF might affect HapT1 tumor angiogenesis. To check this possibility, HapT1 tumor tissues treated with or without MaMIF were microscopically analyzed and the number of blood vessels was quantified. Quantification of vessel density clearly showed a significantly higher number of blood vessels in MaMIF treated tumors than control tumors (Fig. 5D). Together, the data suggest that circulating MIF of non-tumor origin might promote tumor angiogenesis, thereby promoting overall tumor growth *in vivo*.

### Function of intracellular MIF on the overall growth of HapT1 cells *in-vitro*

**Figure 6:**
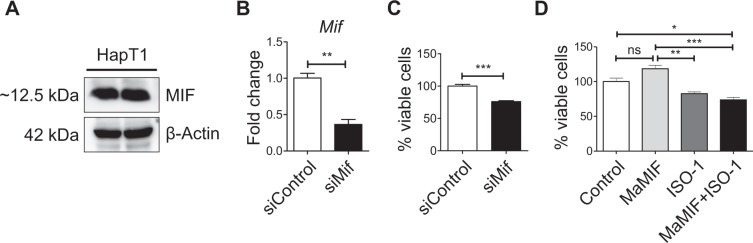
Effect of intracellular MIF on the overall growth of HapT1 cells *in-vitro*. (**A**) Immunoblot analysis showing the MIF expressed by the HapT1 cells (Lysates of two different passages). (**B**) Graph showing the reduced expression of MIF after 48 hours of siRNA transfection (n = 3). Each bar represents the mean ± the SEM. ***p < 0.0001 using a Student t-test. (**C**) Graph showing the percentage of viable cells upon siRNA transfection (n = 3). Each bar represents the mean ± the SEM. **p < 0.001 using a Student t-test. (**D**) Graph showing the percentage of viable cells upon treating with MaMIF (100 ng/ml), ISO-1 (100 µg/ml) and MaMIF + ISO-1 for 48 hours (n = 3). Data represents the mean ± SEM, *p < 0.05; **p < 0.001; ***p < 0.0001 using one-way ANOVA with Bonferroni’s Multiple Comparison Test.

In the aforementioned *in vitro* experiments, exogenous MaMIF treatment did not show any significant effect on HapT1 cells growth. However, this experiment did not rule out the possible role of intracellular MIF on HapT1 cells. To check the significance of intracellular MIF in HapT1 cells, we first confirmed MIF protein expression in these cells (Fig. 6A). After the confirmation of MIF expression, we depleted MIF expression or inactivated its activity in HapT1 cells by siRNA or ISO-1 treatment, respectively. Further cell viability estimation through crystal violet staining after 48 hours of treatment demonstrated that intracellular MIF depletion or inactivation has a significant effect in reducing the overall growth of these cells *in vitro* (Fig. 6B–D).

## Discussion

The cross-reactivity of MIF polyclonal antibody (NBP1-81832; Novus Biologicals**)** generated against a recombinant human MIF having 89% identity with MaMIF indicates conserved antigenic epitopes in human MIF and MaMIF. Moreover, these results confirm the reliability of using NBP1-81832 antibody to detect the MaMIF. The primary sequence and crystal structure analysis of MaMIF showed that mouse and hamster have an almost similar level of identities with human MIF, which indicates that experiments related to MIF in mouse and hamster might have also similar outcomes. However, it will be apt to mention that the overall outcome of an experimental intervention in any animal model depends on multiple factors like the animal’s genetic makeup, physiology, natural behavior, etc. Hence, in certain instances, hamster might be a more clinically relevant model than mice and *vice versa*. For example, hamster is a good host for multiple infectious agents to which mice are resistant^63^. To investigate the role of MIF in the pathogenesis of these diseases, hamster model might be instrumental. Moreover, conserved structural features like the active site for tautomerase and ISO-1 binding in MaMIF further justifies the suitability and relevance of this model in MIF-related studies (Fig. 2D,E, Supplementary Fig. 2). A study has shown that the diameter of the central channel of MIF from parasites like *Giardia lamblia* and *Plasmodium* species is narrower than that of human MIF. This structural difference between human- and parasite-MIF central channels might form a basis for structure-based drug design against the parasites^64^. A wider channel size in human MIF and MaMIF (Fig. 2D) indicates that this structure might have a functional significance in vertebrates, which warrants further investigation.

The K_m_ value of MaMIF protein (Fig. 3C) is not in agreement with the MIF proteins of other species (2.4 mM^65^; 2.7 mM^66^; 2.1 mM^67^). This enzyme was found to have much superior activity, with nearly 4-fold higher sensitivity towards L-dopachrome. This difference might be due to an intrinsic superior enzymatic activity of MaMIF and/or difference associated with the technical process (protein quality, instrument sensitivity, etc.). A further in-depth investigation to compare the tautomerase activity of MaMIF and human MIF, using the same technical procedure might be helpful while exploring for effective MIF-inhibitors. Being a conserved protein and having a unique combination of hormone-, cytokine- and thioredoxin-like properties, MIF is considered as a potent cytokine with pleiotropic effects on immune and inflammatory events^68^. It is known to regulate the expression of pro-inflammatory mediators leading to early patient death in sepsis^69,70^. At the same time, human MIF is known to have a differential effect on the migratory properties of various cell types^52^. The recruitment of immune cells to the site of infection leads to clearance of viral, bacterial and fungal infection but on the other hand, monocytes also contribute to their pathogenesis and inflammation^71^. Effect of MaMIF in augmenting the directional migration of hamster PBMCs towards it shows its chemo-attractant property similar to human MIF (Fig. 3E)^52^. Moreover, the effect of ISO-1 in inhibiting the MaMIF mediated upregulation of the expression of proinflammatory molecules and migration of hamster PBMCs corroborates the information obtained from the structural analysis of MaMIF. These data further suggest that ISO-1 could be reliably used as a MaMIF inhibitor in various *in vitro* and *in vivo* studies.

Due to its remarkable involvement in cytokine cascade in the tumor microenvironment, MIF is described as the connecting link bridging cancer with inflammation^72^ and it is also known to be implicated in angiogenesis in multiple cancer types^73–75^. In the recent past, studies have demonstrated the presence of a high level of MIF in different cancer patients. A high level of MIF is reported from the tumor tissues and circulating blood of pancreatic ductal adenocarcinoma (PDAC) patients^58,59^. The expression level of MIF correlates with poor prognosis of PDAC patients. In most of the studies trying to understand the role of MIF in the progression of different cancers, MIF expression was knocked down in overexpressing cancer cells. However, these attempts do not address the contribution of MIF originating from stromal cells of cancer patients. To best of our knowledge, in the pursuit of understanding the functional role of MIF in pancreatic cancer progression, the current study is the first attempt in which exogenous recombinant MIF has been used to induce high circulating MIF level in pancreatic cancer tumor-bearing animals. The results of this study suggest that MIF of non-cancer cell origin can also promote pancreatic tumor growth. Although HapT1 cells express MIF receptors, the addition of exogenous MIF showing no significant effect on HapT1 cells growth *in vitro* indicates that these cells are less dependent on MIF in a growth-factor enriched culture condition (Fig. 5A,B). A similar kind of effect of MIF has also been reported in mouse 4T1 breast tumor cell line^61^. On the other hand, overexpression of *Vegf* in HapT1 cells upon stimulation with MaMIF and presence of more number of blood vessels in HapT1 tumors suggest a possible mechanism through which circulating MIF might indirectly enhance HapT1 tumor growth by promoting angiogenesis *in vivo*. At the same time, results shown in Fig. 6 suggest that the intracellular MIF in HapT1 cells has a pro-tumorigenic role. This could be due to its interaction with proteins like p53 and Jab1/CSN5 as having been reported earlier^76,77^. Together, the effect of MaMIF in promoting the growth of HapT1 tumors corroborates and confirms the pro-tumorigenic role of MIF in pancreatic cancer progression. Hence, while designing MIF-targeted therapy in pancreatic cancer, all the possible sources of MIF that can affect overall tumor growth need to be taken into consideration.

Multiple studies have used a MIF concentration of 100 ng/ml in different cell culture-based studies^36^. However, MIF expression in various disease conditions is variable and dynamic as MIF is a pathophysiologically inducible factor. The major objective of the current study was to check how MaMIF affects different cell types (immune or cancer) and compare it with the already reported properties of human MIF. Our findings might encourage other investigators to explore the clinical relevance of MaMIF in different contexts by designing studies with different relevant MIF concentrations. Moreover, a better understanding of the mechanisms through which circulating MIF can promote pancreatic tumor progression might provide a rationale to design an effective therapeutic strategy against this deadly disease.

## Conclusion

In the current study, MaMIF has been cloned, expressed and purified for the first time, which will be of significant value for future studies. The crystal structure of MIF will provide direction to study the binding affinity of drugs or inhibitors for therapeutic targeting, which will facilitate experiments on the functional role of MIF in different pathological conditions in a hamster model. As the results of this study show MaMIF to be active both enzymatically and biologically, we believe this work will lead to improved strategies for understanding this molecule in the context of different diseases, including cancer in a hamster model. Importantly, the current study provides convincing experimental evidence which suggests that the hamster model of pancreatic cancer can be reliably used to investigate MIF related questions in pancreatic cancer.

## Supplementary information

Supplementary information
