# Supplementary material for: Macrophage migration inhibitory factor of Syrian golden hamster shares structural and functional similarity with human counterpart and promotes pancreatic cancer
Source: Sci Rep. 2019 Oct 29;9:15507. doi: 10.1038/s41598-019-51947-7 (PMC6820718; doi:10.1038/s41598-019-51947-7)
Supplement: Supplementary file 1 — Supplementary information [file 41598_2019_51947_MOESM1_ESM.pdf]

Macrophage migration inhibitory factor of Syrian golden hamster shares structural and functional similarity with human counterpart and promotes pancreatic cancer

Voddu Suresh[1,3*], Rajivgandhi Sundaram[2,4*], Pujarini Dash[1*], Surendra Chandra Sabat[5], Debasish Mohapatra[1], Sneha Mohanty[6], Dileep Vasudevan[2#], Shantibhusan Senapati[1#]

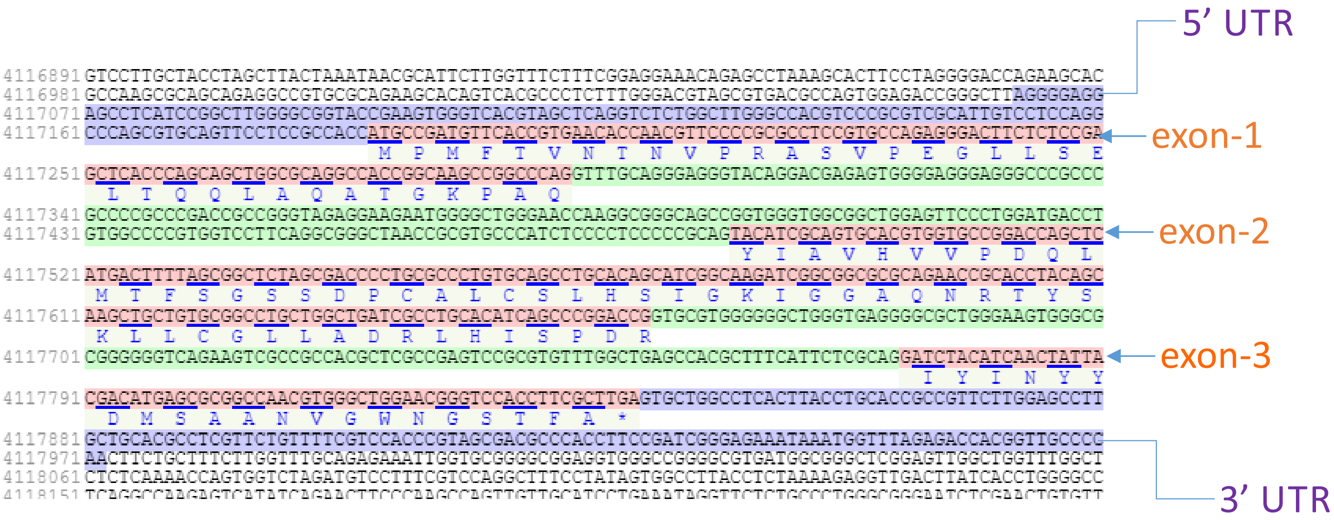

Supplementary Figure 1: Genomic organization of *Mesocricetus auratus* Mif gene shown in NCBI Reference Sequence NW_004801628.1

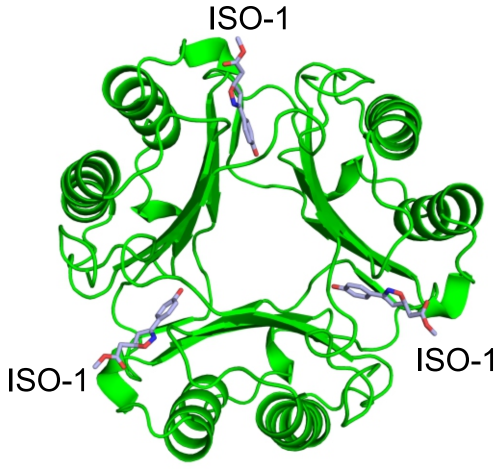

Supplementary Figure 2: Crystal structure of homo-trimeric human MIF (green) in complex with ISO-1 (blue) [PDB id: 1ljt]. The MIF trimer is shown in cartoon representation and the three ISO-1 molecules in stick model.

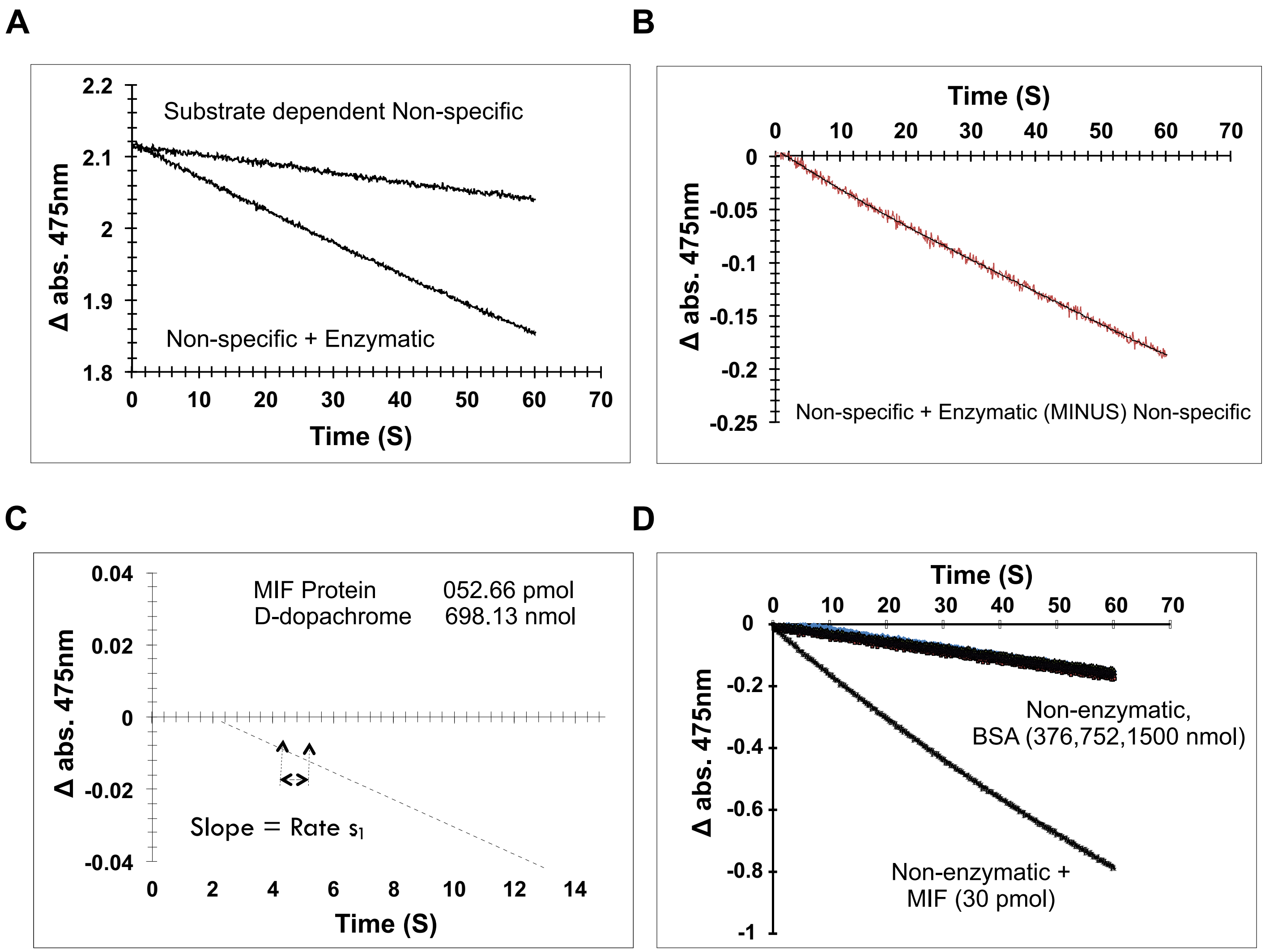

Supplementary Figure 3: Background correction for the non-enzymatic activity of L-dopachrome methyl ester.

Figure 1D & 1E

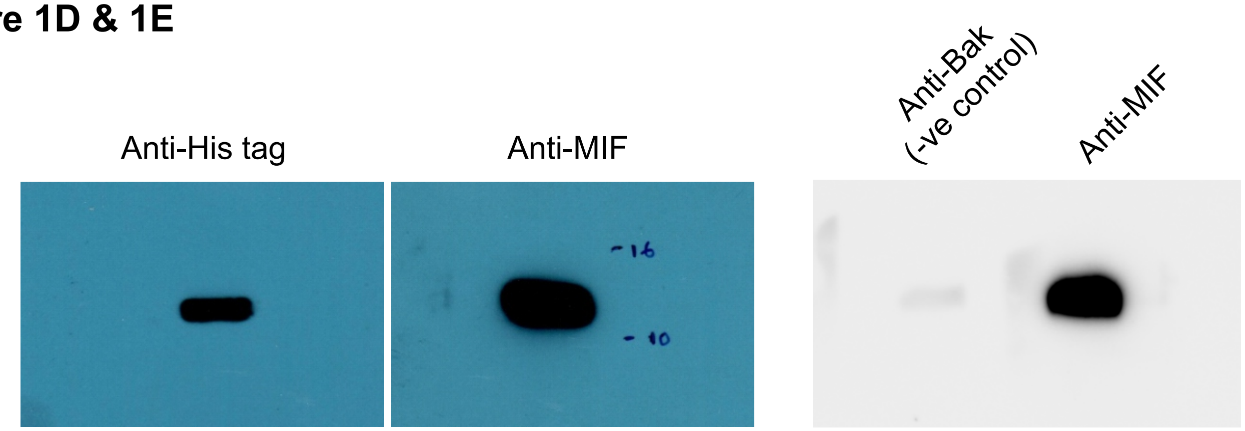

Figure 6 A

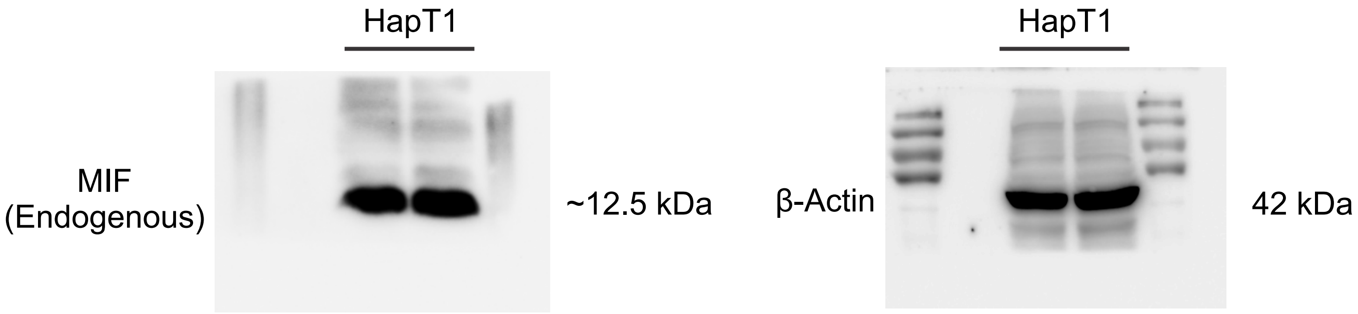

Supplementary Figure 4: Uncropped blots for corresponding figures.
